# The Measurement of the Effect on Citation Inequality of Differences in Citation Practices across Scientific Fields

**DOI:** 10.1371/journal.pone.0058727

**Published:** 2013-03-14

**Authors:** Juan A. Crespo, Yungrong Li, Javier Ruiz–Castillo

**Affiliations:** 1 Departamento de Economía Cuantitativa, Universidad Autónoma de Madrid, Madrid, Spain; 2 Departamento de Economía, Universidad Carlos III de Madrid, Madrid, Spain; Max Planck Society, Germany

## Abstract

This paper has two aims: (i) to introduce a novel method for measuring which part of overall citation inequality can be attributed to differences in citation practices across scientific fields, and (ii) to implement an empirical strategy for making meaningful comparisons between the number of citations received by articles in 22 broad fields. The number of citations received by any article is seen as a function of the article’s scientific influence, and the field to which it belongs. A key assumption is that articles in the same quantile of any field citation distribution have the same degree of citation impact in their respective field. Using a dataset of 4.4 million articles published in 1998–2003 with a five-year citation window, we estimate that differences in citation practices between the 22 fields account for 14% of overall citation inequality. Our empirical strategy is based on the strong similarities found in the behavior of citation distributions. We obtain three main results. Firstly, we estimate a set of average-based indicators, called *exchange rates*, to express the citations received by any article in a large interval in terms of the citations received in a reference situation. Secondly, using our exchange rates as normalization factors of the raw citation data reduces the effect of differences in citation practices to, approximately, 2% of overall citation inequality in the normalized citation distributions. Thirdly, we provide an empirical explanation of why the usual normalization procedure based on the fields’ mean citation rates is found to be equally successful.

## Introduction

The field dependence of reference and citation counts in scientific articles in the periodical literature has been recognized since the beginning of Scientometrics as a field of study (see *inter alia* [1]–[3]). There are multiple reasons. Consider the differences across scientific disciplines in, for example, (i) size, measured by the number of publications in the periodical literature; (ii) the average number of authors per paper; (iii) the average paper length; (iv) the average number of papers per author over a given period of time; (v) the theoretical or experimental mix that characterizes each discipline; (vi) the average number of references per paper; (vii) the proportion of references that are made to other articles in the periodical literature; (viii) the percentage of internationally co-authored papers, or (ix) the speed at which the citation process evolves.

Given a classification of science into scientific disciplines, this paper develops a measuring framework where it is possible to quantify the importance of differences in citation practices. We use a model in which the number of citations received by an article is a function of two variables: the article’s underlying scientific influence, and the field to which it belongs. In this context, the citation inequality of the distribution consisting of all articles in all fields -the all-fields case- is the result of two forces: differences in scientific influence, and differences in citation practices across fields. The first aim of the paper is how to isolate the citation inequality attributable to the latter, and how to measure its importance relative to overall citation inequality of all sorts.

The first difficulty we must confront is that the characteristics of the scientific influence distributions are *a priori* unknown. Thus, even if they were observable, we would not know how to compare the scientific influence of any two articles belonging to different fields. To overcome this difficulty, we make the strong assumption that articles in the same quantile of the scientific influence distribution have the same degree of scientific influence independently of the field to which they belong. Thus, if your article and mine belong, for example, to the 80th percentile of our respective scientific influence distributions, then we assume that they have the same degree of scientific influence.

The next difficulty is that scientific influence is an unobservable variable. To overcome this difficulty, we assume that, given the field, citation impact varies monotonically with scientific influence. Thus, if one article has greater scientific influence than another one in the same field, then we expect the former to have also a greater citation impact than the latter. The monotonicity assumption ensures that, for any field, the quantiles of the (unobservable) scientific influence distribution coincide with the quantiles of the corresponding (observable) citation distribution. Therefore, if the mean citation of articles in, for example, the 80th percentile of your field is twice as large as the mean citation of articles in the same percentile in my field, this means that your field uses twice the number of citations as mine to represent the same degree in scientific influence. The implication is that the citation inequality of the set of articles in each field belonging to the same quantile can be solely attributed to idiosyncratic differences in citation practices across fields. Thus, the aggregation of this measure over all quantiles provides a method for quantifying the effect of these differences (This is, essentially, John Roemer’s [4], model for the study of inequality of opportunities in an economic or sociological context).

Following [5], we implement this model by using an additively decomposable inequality index, in which case the citation inequality attributed to differences in citation practices is captured by a between-group inequality term in the double partition by field and citation quantile. For our purposes, it would be ideal that the scientific community would have agreed upon a classification of science into a number of disciplines. Unfortunately, there are many different classification systems (see [6] for a recent attempt of building a classification system, as well as a review of the present situation). For expository reasons, in this paper we choose a very simple classification system into 22 broad fields distinguished by Thomson Reuters. Specifically, using a dataset of 4.4 million articles published in 1998–2003 with a five-year citation window and an appropriate citation inequality index, we estimate that the citation inequality attributable to differences in citation practices across the 22 fields represents, approximately, 14% of overall citation inequality (in a companion paper, [7], we extend the analysis to the 219 Web of Science subject categories created by the same firm).

It would appear that, regardless of how their impact can be measured, differences in publication and citation practices pose insurmountable obstacles to direct comparisons of the absolute number of citations received by articles in different fields. For example, in the dataset used in this paper, how can we interpret the fact that the mean citation in Mathematics is 2.4, about eight and a half times smaller than in Molecular Biology and Genetics where it is equal to 20.4 citations? This paper shows that the striking similarity between citation distributions (documented at different aggregation levels in [8], [9] and [10]), causes the citation inequality attributable to different citation practices to be approximately constant over a wide range of quantiles. This allows us to estimate a set of average-based indicators, which we call exchange rates, that serve to answer the following two questions. Firstly, how many citations received by an article in a given field are equivalent to, say, 10 citations in the all-fields case? For example, in Clinical Medicine the answer is 12.1 with a standard deviation (StDev hereafter) of 0.6, while in Engineering the answer is 4.4 with a StDev of 0.2. Secondly, how much can we reduce the effect of different citation practices by normalizing the raw citation data with the exchange rates? We find that this normalization procedure reduces this effect from 14% to, approximately, 2% of overall citation inequality.

The difficulty of comparing citation counts across scientific fields is a very well known issue that has worried practitioners of Scientometrics since its inception. Differences in citation practices are usually taken into account by choosing the world mean citation rates as normalization factors (see *inter alia* [11]–[21]). More recently, other papers support this traditional procedure on different grounds ([10], [22], [23]). In our last contribution, we find that using field mean citations as normalization factors leads to a slightly greater reduction of the effect of differences in citation practices on citation inequality than our exchange rates. We show how our model helps explaining why the traditional model is so successful.

Methods that use mean citations or exchange rates as normalization factors belong to the class of target or “cited side” normalization procedures. Following an idea in [24], source or “citing side” procedures have been recently suggested (see *inter alia* [25]–[30]). Since our dataset lacks citing side information, applying this type of procedure is beyond the scope of this paper. On the other hand, it should be emphasized that the conceptual and empirical approaches developed in this paper for the all-sciences case, can be equally applied to a situation in which articles belonging to a number of closely related but heterogeneous sub-fields need to be aggregated into a single intermediate category, such as the aggregation of Organic Chemistry, Inorganic Chemistry, Chemical Engineering and other sub-fields into the discipline “Chemistry”.

The rest of the paper consists of three Sections. Section 2 introduces the model for the measurement of the effect of differences in citation practices. Section 3 presents the estimation of average-based exchange rates and their StDevs over a long quantile interval. It also discusses the consequences of using such field exchange rates and mean citations as normalization factors. Section 4 contains some concluding comments.

## The Model

### 1.1 Notation and Comparability Conditions

From an operational point of view, a scientific field is a collection of papers published in a set of closely related professional journals. In this paper, we take as *a priori* given a classification system consisting of 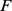 fields, indexed by 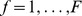. Let 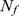 be the total number of articles in field 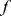, and let 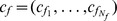 be the citation distribution for that field where, for each 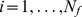, 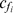 is the number of citations received by the 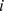-th article. The total number of articles in the all-fields case is 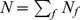. The number of citations of any article, 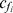, is assumed to be a function of two variables: the field 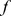 to which the article belongs, and the scientific influence of the article in question, 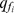, which is assumed for simplicity to be a single-dimensional variable. Thus, for every 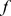 we write:
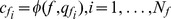
(1)


Let 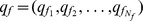 with 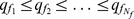 be the ordered distribution of scientific influence in every field. It is important to emphasize that distribution 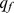 is assumed to be a characteristic of field 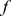. Furthermore, no restriction is imposed *a priori* on distributions 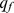, 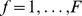. Consequently, for any two articles 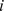 and 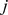 in two different fields 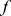 and 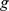, the values 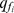 and 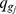 cannot be directly compared. To overcome this difficulty, in this paper we introduce some structure into the comparability problem by means of the following key assumption.

#### Assumption 1 (A1)

*Articles at the same quantile 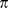 of any field scientific influence distribution have the same degree of scientific influence in their respective field.*

Typically, scientific influence is an unobservable variable. However, although the form of 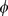 in Eq. 1 is unknown, we adopt the following assumption concerning it:

#### Assumption 2 (A2)

*The function* 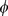 *in expression 1 is assumed to be monotonic in scientific influence, that is, for every pair of articles 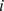 and 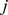 in field* 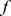, *if* 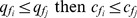.

Under A2, the degree of scientific influence uniquely determines the location of an article in its field citation distribution. In other words, for every 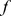, the partition of the scientific influence distribution 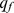 into 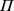 quantiles of size 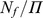, 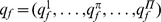, induces a corresponding partition of the citation distribution 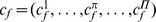 into 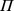 quantiles, where 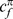 is the vector of the citations received by the 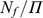 articles in the 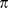-th quantile of field 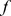. Assume for a moment that we disregard the citation inequality within every vector 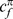 by assigning to every article in that vector the mean citation of the vector itself, namely, 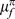. Since the quantiles of citation impact correspond –as we have already seen– to quantiles of the underlying scientific influence distribution, holding constant the degree of scientific influence at any level as in A1 is equivalent to holding constant the degree of citation impact at that level. Thus, the interpretation of the fact that, for example, 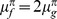 is that, on average, field 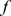 uses twice the number of citations as field 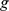 to represent the same underlying phenomenon, namely, the same degree of scientific influence in both fields. Hence, for any 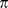, the difference between 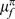 and 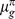 for articles with the same degree of scientific influence is entirely attributable to differences in citation practices between the two fields.

Welfare economists would surely recognize the above as Roemer’s [4] model for the inequality of opportunities where individual incomes (or other indicators of performance, such as educational outcomes) are assumed to be a function of two types of factors: a set of variables outside an individual’s responsibility – the *circumstances*, mainly inherited from our parents–, and *effort*, an unobservable single dimensional variable entirely within the sphere of each individual’s responsibility. Which are the relevant circumstances is a difficult philosophical and political problem, whose solution is typically affected by the availability of information in practical situations. Be it as it may, the *a priori* given circumstances determine a partition of the population into *types*. In this model, income inequality holding constant the degree of effort by every type is seen to be entirely due to differences in circumstances, or to the *inequality of opportunities* at this degree of effort. According to Roemer, income inequality due to differences in effort is not worrisome from a social point of view. It is income inequality due to differences in circumstances, namely, the inequality of opportunities, what society might attempt to compensate for. Individuals are articles; the equivalent of income is citations; the *a priori* given partition of individuals into types is equivalent to the *a priori* given classification system of articles into fields; effort is scientific influence; and the inequality of opportunities is the citation inequality attributable to differences in citation practices.

### 1.2 The Measurement of the Effect of Differences in Citation Practices

Given a classification system, let 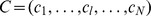 be the overall citation distribution in the all-fields case, where, for each 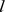, there exists some article 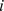 in some field 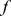 such that 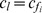. To develop our measurement framework, it is convenient to work with additively decomposable citation inequality indices. For any partition of the population into subgroups, an additive decomposable citation inequality index allows to express the overall citation inequality as the sum of two terms: a within-group term, which is the weighted sum of the citation inequality within all subgroups, and a between-group term,which is equal to the citation inequality of a distribution where every article is assigned the mean citation of the subgroup to which it belongs. In the income inequality literature it is well known that the so-called Generalized Entropy family of inequality indices are the only measures of income inequality that satisfy the usual properties required from any inequality index and, in addition, are decomposable by population subgroup ([31]–[33] ). In this paper we choose a certain member of this family, denoted by 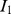, and defined as:
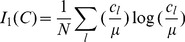
(2)where 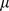 is the mean of distribution 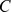 (To solve the problem of 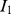 not being defined for articles without citations we have followed the convention 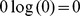. For a discussion of the robustness of our results to different conventions see the working paper version of this paper [34]). The main reason for selecting 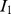 is that, for any partition, the weights in the within-group term in this index decomposable form are the subgroups’ citation shares. Thus, in the partition of a citation distribution into quantiles that will play a key role in what follows, the higher the quantile, the greater is the weight attributed to it. Within the Generalized Entropy family, the natural alternative would be to choose an index 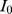 in which these weights are the subgroups’ demographic shares. In the example of the partition into citation quantiles, all quantiles will be equally weighted. In our context, given the skewness of citation distributions (see *inter alia* [8], [9]), we believe that the option we have taken is clearly preferable.

Using the additive decomposability property of 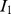, it can be shown that the overall citation inequality in the double partition of distribution 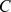 into 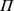 quantiles and 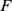 fields can be expressed as the sum of the following three terms:
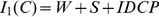
(3)where:



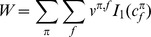


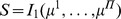


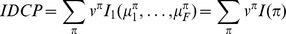
where 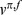 is the share of total citations in quantile 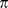 of field 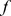 and 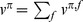.

The term 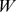 is a within-group term that captures the weighted citation inequality within each quantile in every field. Obviously, since all articles in each vector 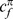 belong to the same field, there is no difficulty in computing the expression 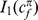. Note that, for any 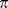, if for two fields 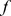 and 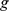 we have 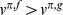, then the citation inequality within the vector 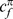 will carry more weight in the term 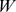 than the citation inequality within the vector 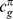. However, for large 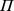, 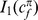 is expected to be small for all 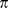 and all 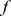. Thus, the weighting issue will be relatively unimportant, and the term 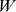 as a whole is also expected to be small.

The term 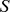 is the citation inequality of the distribution 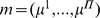 in which each article in a given quantile 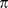 is assigned the quantile’s citation mean, 
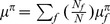
. Thus, 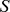 is a measure of citation inequality at different degrees of citation impact that captures well the skewness of science in the all-fields case. Due to the high skewness of all citation distributions, the term 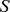 is expected to be large.

Finally, for any 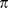, the expression 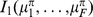, abbreviated as 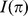, is the citation inequality attributable to differences in citation practices according to 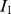. Thus, the weighted average that constitutes the third term in expression 3, denoted by 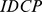 (*I*nequality due to *D*ifferences in *C*itation *P*ractices), provides a good measure of the citation inequality due to such differences. Note that, for any 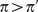, 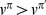. Thus, as indicated before, higher quantiles carry more weight than lower quantiles in the crucial 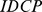 term. Due to the skewness of science, this effect is expected to give a very large role to the citation inequality attributable to differences in citation practices at the upper tail of citation distributions.

In this paper only research articles or, simply, articles, are studied. Our dataset consists of 4.4 million articles published in 1998–2003, and the 35 million citations they receive after a common five-year citation window for every year. We study the case where each article is assigned to only one of the 20 broad fields in the natural sciences and the two fields in the social sciences distinguished by Thomson Reuters. Given the heterogeneous composition of at least some of these broad fields, it must be recognized that adopting assumption 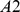 is not very realistic. Consider two publications 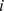 and 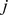 in the same field that belong to two sub-fields with a rather different citation density. Contrary to 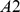, it may be very well the case that article 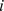 has greater scientific influence but receives less citations than article 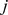. Lower aggregation levels would ensure greater homogeneity within sub-fields. However, in the Thompson Reuters system, we would have to face the complication that many articles are assigned to two or more sub-fields (see [35] for a discussion). Therefore, in this introductory paper we will keep working with the 22 fields just introduced (Table A in the Appendix in [34], presents the number of articles and mean citation rates by field).

In this scenario, when 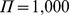, the estimates of all terms in expression 3 are the following:
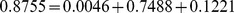



As expected, the term 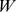 is small, while the term 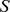 is large, representing 0.52% and 85.53% of overall citation inequality. Consequently, the 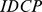 term represents 13.95% of the total citation inequality (see [34] for the robustness of this result for the alternatives 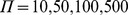).

## Comparability and Normalization Results

This Section analyzes two empirical problems: (i) how to compare the citations received by two articles in any pair of the 22 fields in our dataset by using what we call exchange rates, and (ii) how much the effect of differences in citation practices is reduced when these exchange rates, or the field mean citations are used as normalization factors.

### 2.1 The Comparison of Citation Counts Across Different Fields

How can we compare the citation counts across different fields at a given quantile 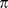
? Recall that the mean citation of articles belonging to field 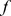 and quantile 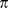 is denoted by 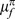, while the mean citation of articles in that quantile is denoted by 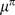. To express the citations in any field in a given quantile in terms of the citations in a reference situation, we find it useful to define the *exchange rates at quantile* 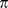, 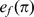, by
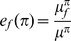
(4)


In the metaphor according to which a field’s citation distribution is like an income distribution in a certain currency, the exchange rates 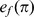 permit to express all citations for that 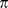 in the same reference currency: since 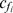 is the number of citations received by article 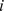 in quantile 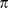 of field 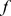, the ratio 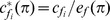 is the equivalent number of citations in the reference currency at that quantile.

Suppose that, for many fields, the exchange rates 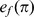 vary drastically with 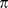. Then we might not be able to claim that differences in citation practices have a common element that can be precisely estimated. However, we next establish that exchange rates are sufficiently constant over a wide range of quantiles.

**Figure 1:**
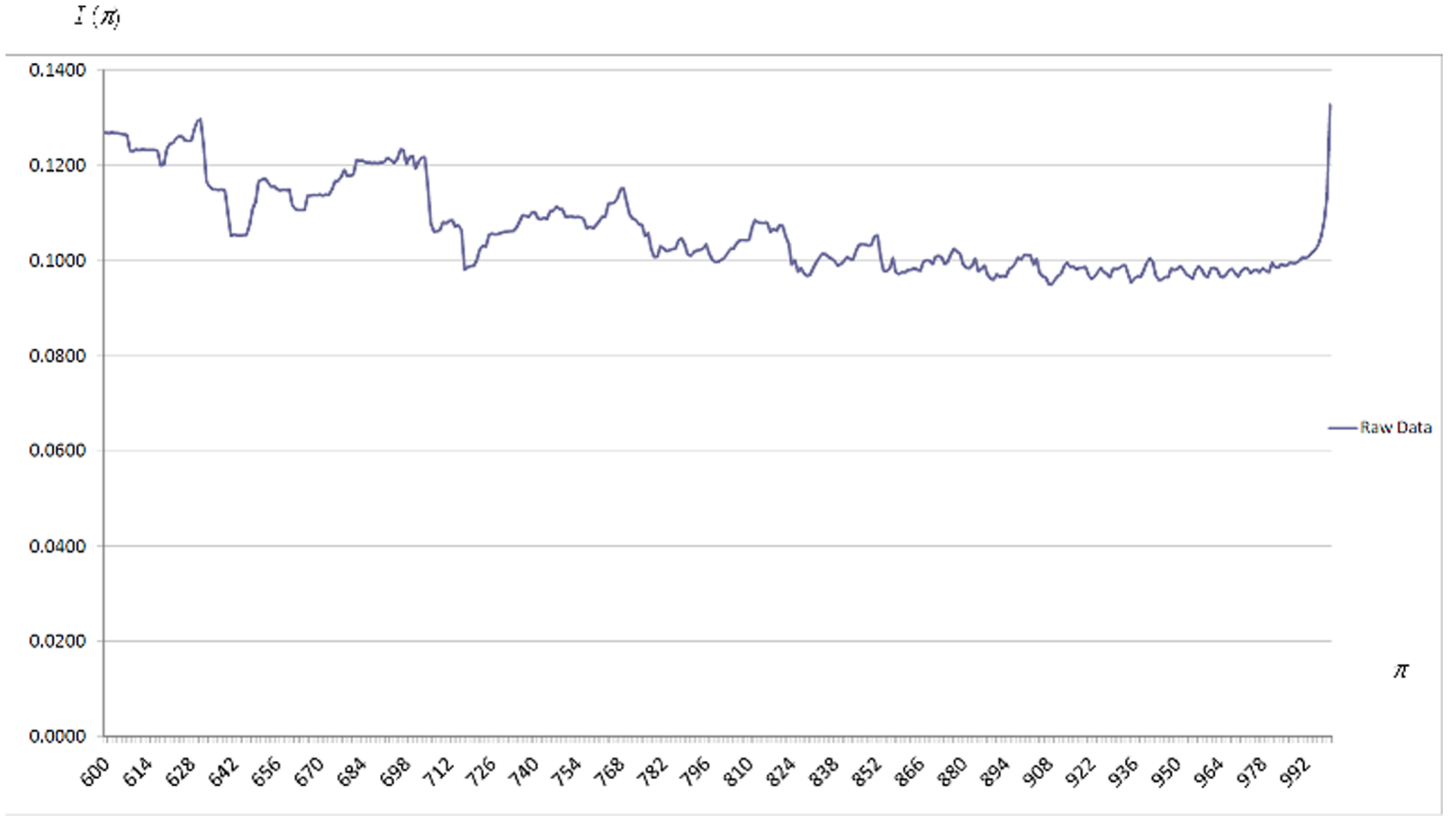
Citation Inequality Due to Differences in Citation Practices, 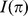, as a function of 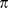. Raw data. Results for the 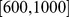 quantile interval.

The effect of differences in citation practices at a given quantile is measured by the expression 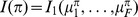 introduced above. It is very instructive to have a graphical representation in Figure 1 of how 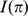 changes with 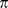 when 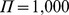 (since 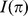 is very high for 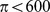, for clarity these quantiles are omitted from Figure 1. It is observed that 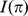 is particularly high until 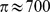, as well as for a few quantiles at the very upper tail of citation distributions. However, 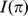 is strikingly similar for a wide range of intermediate values. It is important to emphasize that this is consistent with the stylized facts characterizing citation distributions documented in [8] and [9] using a scale- and size-independent approach: although the percentages of articles belonging to three broad classes are very similar across fields, citation distributions are rather different in a long lower tail and at the very top of the upper tail.

In this situation, it is reasonable to define an *exchange rate* (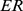 hereafter) over some interval 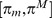 in that intermediate range as the arithmetic mean of the exchange rates (defined in Eq. 4) for every quantile in that interval:
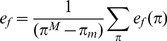
(5)


An advantage of this definition is that we can easily compute the associated StDev, denoted by 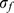. The fact that, for each 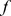, the 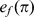 defined in 4 are very similar for all 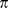 in the interval 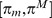 would manifest itself in a small 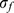, and hence in a small coefficient of variation 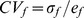. We find that the choice 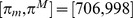 – where 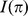 for most 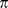 is equal to or smaller than 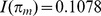 and 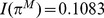 – is a good one. The 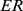
s 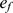, as well as the 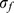, and 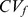 are in columns 1 to 3 in Table 1. For convenience, 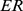
s are multiplied by 10. Thus, for example, the first row indicates that 15.8 citations with a StDev of 0.9 for an article in Biology and Biochemistry between, approximately, the 71st and the 99th percentile of its citation distribution, are equivalent to 10 citations for an article in that interval in the all-fields case.

**Table 1:** Exchange Rates, Standard Deviations, and Coefficient of Variation for the 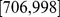 Interval, and Exchange Rates Based on Mean Citations.

	ExchangeRates	StandardDeviation	Coefficientof Variation	% ofCitations	*ER*s based onMean Citations	*ER*s based onMean Cits.in the [706,998]interval
	(1)	(2)	(3)	(4)	(5)	(6)
1. Biology & Biochemistry	15.8	0.9	0.054	68	16	15.3
**2. Clinical Medicine**	12.1	0.6	**0.049**	71.8	12.4	12.5
**3. Immunology**	19.5	0.9	**0.048**	66.3	20.4	19
4. Microbiology	14.4	1.3	0.092	65.8	14.6	13.5
**5. Molecular Biology & Genetics**	25.7	0.6	**0.022**	71.1	25.9	25.9
**6. Neuroscience & Behav. Science**	17.1	0.8	**0.050**	67.2	17.5	16.5
7. Pharmacology & Toxicology	10.1	0.6	0.056	68.4	10.2	9.8
**8. Psychiatry & Psychology**	9.1	0.2	**0.025**	72.4	9	9.1
**9. Chemistry**	9.9	0.4	**0.037**	70.9	9.7	9.7
10. COMPUTER SCIENCE	3.7	0.5	*0.124*	76.3	3.8	4
11. Mathematics	3.3	0.2	0.059	75.4	3.1	3.3
12. Physics	8.8	0.5	0.061	74.2	8.7	9.1
**13. Space Science**	14.2	0.3	**0.019**	71.9	14	14.2
14. Agricultural Sciences	6.5	0.4	0.056	72.5	6.2	6.3
15. Engineering	4.4	0.2	0.054	75.9	4.1	4.4
16. Environment & Ecology	9.1	0.7	0.073	68.3	9.1	8.7
17. Geoscience	8.9	0.6	0.069	70.1	8.6	8.5
**18. Materials Science**	5.9	0.3	**0.048**	75	5.8	6.1
19. MULTIDISCIPLINARY	4.3	0.7	*0.158*	81.6	4.1	4.7
**20. Plant & Animal Science**	6.7	0.3	**0.045**	71.3	6.5	6.5
21. Economics & Business	5.2	0.4	0.068	75.6	5	5.3
**22. Social Sciences, General**	4.5	0.2	**0.045**	75.1	4.2	4.5
**Mean**				**72.1**		

As a referee has pointed out, the approach discussed in the recent scientometrics literature on percentile-based indicators (see *inter alia* [36]–[38]) seems to follow in a natural way from our assumptions 1 and 2. Under this approach, the following type of ordinal comparison is justified. Assume that, in spite of the fact that your paper receives 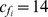 citations in field 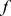 and mine receives 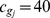 in field 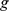, paper 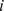 belongs to the 80th percentile in field 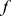 while paper 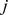 belongs to the 60th percentile in field 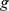. Then, we can conclude that your paper has a greater degree of scientific influence than mine. By exploiting the fact that citation distributions seem to differ only by a scale factor over a large quantile interval in which 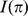 remains essentially constant, what this paper adds is the possibility of establishing cardinal comparisons of the following type. Assume that the 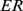
s are 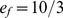 and 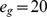, so that the normalized citations are 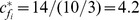, and 
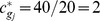
. Since 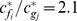, we can conclude that your paper has a degree of scientific influence that is approximately twice as great as mine.

We find it useful to divide fields into three groups according to the 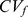. Group I (bold letters in Table 1), consisting of 10 fields, has a 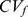 smaller than or equal to 0.05. This means that the StDev of the exchange rate, 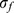, is less than or equal to five percent of the exchange rate itself. Hence, we consider 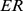
s in this group as highly reliable. Group II (regular type), consisting of 10 fields, has a 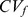 between 0.05 and 0.10. We consider the 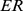
s in this group to be fairly reliable. Group III (capital letters), consists of two fields: Computer Science, with a 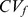 greater than 0.10, which is known from previous work to behave as an outlier ([35] ), and the Multidisciplinary field with a 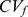 greater than 0.15, a hybrid field that does not behave well either in [10]. The results for these two fields should be considered unreliable.

As is observed in the last row of column 4 in Table 1, the mean of the percentage of citations covered by the interval 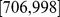 in the 22 fields is 72.1% (with a StDev of 3.9). Although this is a large percentage, expanding the interval in either direction would bring a larger percentage of citations. It turns out that the 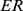
s do not change much. However, they exhibit greater variability (for details, see [34]). Therefore, we find it useful to retain the interval 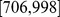 in the sequel.

### 2.2 Normalization Results

Given a classification system, citation inequality due to differences in scientific influence –captured by the 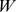 and 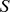 terms in Eq. 3– poses no problem. Instead, we would like to eliminate as much as possible the citation inequality attributable to differences in citation practices within that system. Thus, the impact of any normalization procedure can be evaluated by the reduction in the term 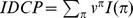 in Eq. 3 before and after normalization.

**Figure 2:**
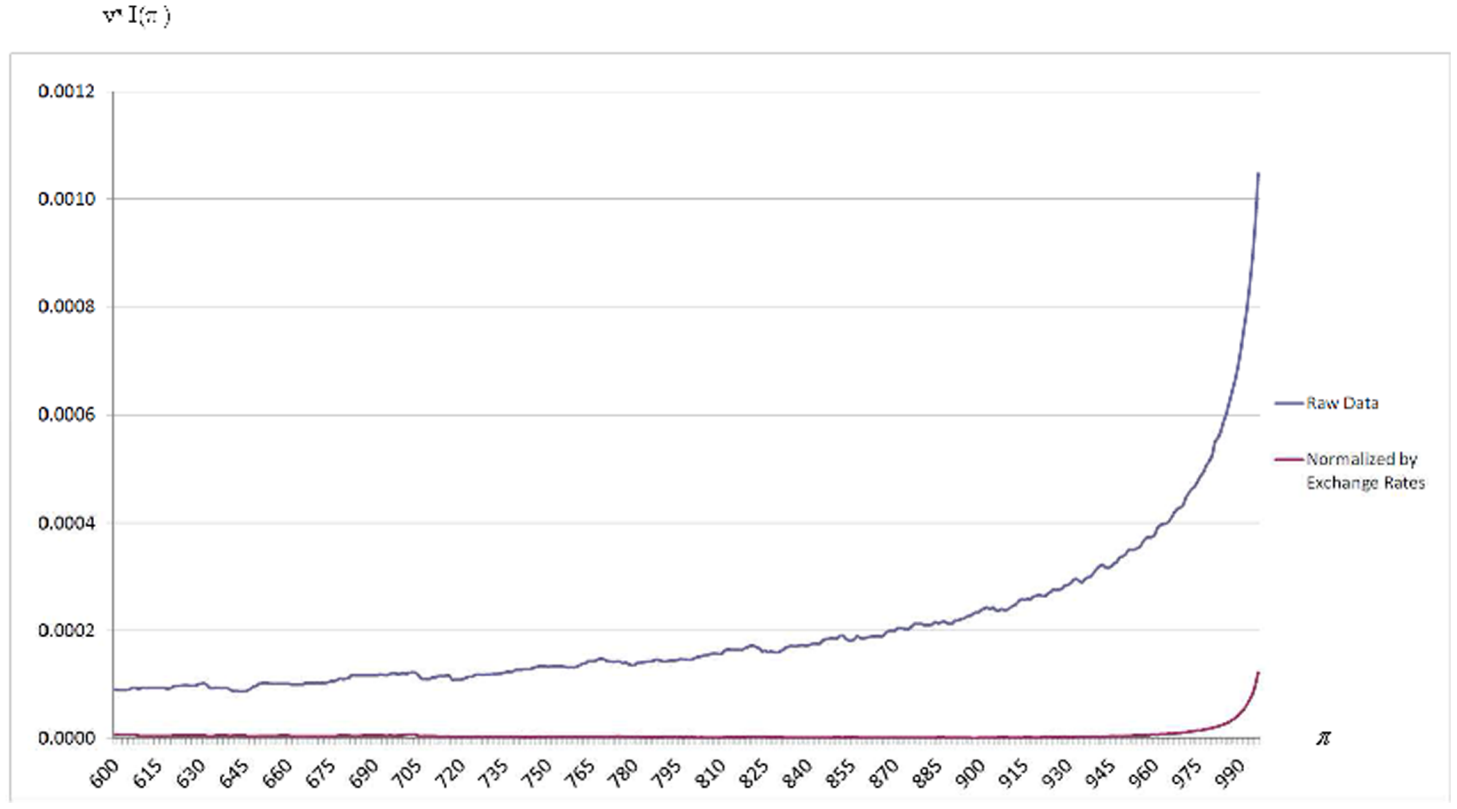
Weighted Citation Inequality Due to Differences in Citation Practices, 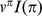, as a function of 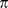. Raw vs. Normalized data. Results for the 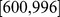 quantile interval.

Figure 2 focuses on the product 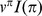 as a function of 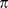. Of course, the term 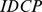 is equal to the integral of this expression (for clarity, quantiles 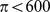, and 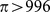, are omitted from Figure 2. The skewness of science causes the weights 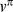 to be very small for a large initial quantile interval, but rapidly increasing as we proceed towards higher quantiles. Note the strong impact of this weighting system on the shape of the 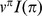 curve when we use the raw data in the blue curve. On the other hand, relative to the blue curve the red curve illustrates the correction achieved when we use the exchange rates in Table 1 as normalization factors: the size of the 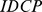 term is very much reduced. The numerical results before and after this normalization are in Panels A and B in Table 2.

**Table 2:** Total Citation Inequality Decomposition Before and After Normalization: 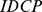 Interval Detail.

	Within-group	Skew. of Sc.	*IDCP*	Total Citation	Percentages in %:		
	Term, *W*	Term, *S*	Term	Ineq., *I*_1_(*C*)	(1)/(4)	(2)/(4)	(3)/(4)
	(1)	(2)	(3)	(4)	(5)	(6)	(7)
**A. RAW DATA**							
**All Quantiles**	0.0046	0.7488	0.1221	0.8755	0.53	85.52	13.95
**[1, 705]**			0.0449				5.13
**[706, 998]**			0.0717				8.18
**[999, 1000]**			0.0056				0.64
**B. EXCHANGE RATE**							
**NORMALIZATION**							
**All Quantiles**	0.0051	0.7788	0.0167	0.8006	0.63	97.28	2.09
**[1, 705]**			0.0127				1.59
**[706, 998]**			0.0018				0.23
**[999, 1000]**			0.0022				0.27
**C. MEAN**							
**NORMALIZATION**							
**All Quantiles**	0.005	0.7794	0.0164	0.8008	0.63	97.32	2.05
**[1, 705]**			0.0124				1.55
**[706, 998]**			0.002				0.25
**[999, 1000]**			0.002				0.25

Note that both the 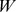 and the 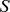 terms remain essentially constant after normalization. However, the 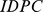 term is reduced from 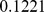 to 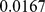, an 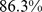 difference. Of course, total citation inequality after normalization is also reduced. On balance, the 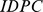 term after normalization only represents 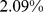 of total citation inequality – a dramatic reduction from the 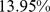 with the raw data. However, it should be recognized that in the last two quantiles and, above all, in the 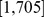 interval normalization results quickly deteriorate. The problem is that citation inequality due to different citation practices in that interval is both high and extremely variable for different quantiles. We have explored the possibility of computing the 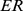
s according to Eq. 5 for the entire 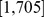 interval. However, this leads to a worsening of the situation. On the other hand, the improvement achieved with a second set of 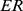
s restricted to the interval 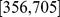 is, at most, very slight (see [34]).

As indicated in the Introduction, the difficulties of combining heterogeneous citation distributions into broader aggregates have been traditionally confronted using the field mean citations as normalization factors (see [34] for a review of this literature). In our dataset, the 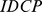 term after the traditional normalization procedure only represents 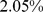 of total citation inequality (see Panel C in Table 2). The two solutions are so near that we refrain from illustrating the latter in Figure 2 because it will be indistinguishable from the red curve after normalization by our ERs. This confirms the results in Radicchi and Castellano [10] where it is concluded that the traditional solution provides a very good approximation to the results obtained with their own procedure for making citation counts independent of the scientific field using a two-parameter transformation.

The question is, how can this similarity of results be accounted for? The explanation is as follows. As documented in [9], field mean citations 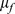 are reached, on average, at the 69.7 percentile with a StDev of 2.6, that is, at the lower bound of our 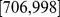 interval. Thus, the 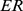
s based on mean citations, 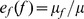 (reproduced in column 5 in Table 1), are approximately equal to our own 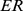
s (in column 1 in that Table). In other words, let 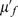 and 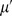 be the mean citations in each field and the population as a whole restricted to the 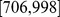 interval, and consider the average-based 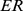
s based on these restricted means: 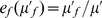 (see column 6 in Table 1). Since field citation distributions differ approximately by a set of scale factors only in the interval 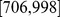, these scale factors should be well captured by any average-based measure of what takes place in that interval – such as our own 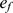, or the new 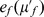. However, the latter 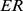
s are essentially equal to the old ones, that is, for each 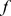, 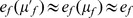.

### Conclusions

The lessons that can be drawn from this paper can be summarized in the following five points.

1. Given a classification system, we have provided a simple method for the measurement of the effect of differences in citation practices across scientific fields. Using a member of a family of additively separable citation inequality indices, this effect is well captured by a between-group term – denoted 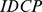 – in the double partition by field and quantile of the overall citation distribution in the all-fields case. It should be noted that this is a distribution free method, in the sense that it does not require that the scientific influence or the citation distributions satisfy any specific assumptions. Using a large dataset of 4.4 million articles in 22 scientific fields and a five-year citation window, we have estimated that the 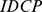 term represents about 14% of overall citation inequality – a result which is independent of the number of quantiles.
2. The striking similarity of citation distributions allows the effect of idiosyncratic citation practices to be rather well estimated over a wide range of intermediate quantiles where citation distributions seem to differ by a scale factor. Consequently, a set of 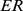
s has been estimated in the interval 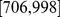 for two purposes: the comparison of the citations received by articles in different fields within that interval, and the normalization of the raw citation data for aggregation purposes. Such 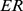
s are estimated with a reasonably low StDev for 20 out of 22 fields.
  It should be stressed that, for uncited and poorly cited articles below the mean, and for articles at the very top of citation distributions, no clear answer to the comparability of citation counts for articles in different fields can be provided. Since the citation process evolves at a different velocity in different fields, using variable citation windows to ensure that the process has reached a similar stage in all fields should improve field comparability at the lower tail of citation distributions. Naturally, we may also worry about how to compare citation counts in the last two quantiles of citation distributions. Given the fact that in this key segment the citation impact appears to be very diverse across fields, perhaps this task should not even be attempted. Until we know more concerning how differential citation practices operate in these top quantiles, the most we can do within this paper’s framework is to use 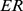
s 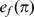 for 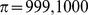.
3. Given a classification system, the success of any normalization procedure in eliminating as much as possible the impact of differences in citation practices can be evaluated by the reduction it induces in the 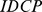 term. In our case, it has been established that both the procedure that uses our 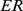
s, as well as the traditional method of taking the field citation means as normalization factors reduces the importance of the 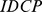 term relative to overall citation inequality from, approximately, 14% to 2%. The paper provides an empirical explanation of why the two methods are equally successful. Finally, as explained in [34], the normalization advocated by Glanzel [39] reduces the 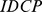 term to 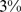 of overall citation inequality.
  Other normalization proposals – such as the one in Radicchi and Castellano [10], or those based on “citing” side procedures quoted in the Introduction – might be analogously evaluated. In turn, it would be interesting to evaluate the normalization procedure based on the 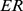
s in terms of the reduction of the bias in the Radicchi and Castellano [10] model. Given how near our 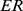
s are to those based on the fields’ mean citation rates, the conjecture is that our procedure would perform as well as the approximation provided by these means in Radicchi and Castellano.
4. It should be emphasized that the method for quantifying the importance of differences in citation practices before and after a normalization procedure takes as given a certain classification system. Thus, the greater the number of fields distinguished, the greater is the percentage that the 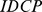 term is expected to represent relative to overall citation inequality. More importantly, normalization procedure 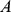 may be more effective than normalization procedure 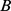 for a certain classification system, but the opposite may be the case for another one.
  As indicated in the Introduction, in a companion paper [7] we have used the same dataset at a lower aggregation level with 219 sub-fields identified with the Web of Science subject categories. The following three findings should be emphasized. Firstly, in the presence of 219 sub-fields the 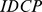 term represents about 18% of overall citation inequality. Secondly, the coefficient of variation of 187/190 sub-fields out of the total 219 are smaller than or equal to 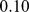. Thirdly, using the 219 exchange rates or the 219 field mean citations as normalization factors reduces the importance of the 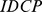 term to 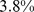 and 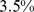, respectively.
5. Naturally, policy makers and other interested parties should be very cautious when comparing citation performance in different scientific fields. More research is still needed. In particular, we need to study the robustness of our strategy to datasets from other periods, other sources – such as Scopus –, and other classification systems. However, together with the important contribution by Radicchi and Castellano [10] and the works on “citing side” procedures, the results of this paper indicate that the combination of interesting assumptions with the empirical similarity of citation distributions paves the way for meaningful comparisons of citation counts across heterogeneous scientific disciplines.
