# Correction: The Measurement of the Effect on Citation Inequality of Differences in Citation Practices across Scientific Fields

**DOI:** 10.1371/annotation/d7b4f0c9-8195-45de-bee5-a83a266857fc

**Published:** 2013-05-07

**Authors:** Juan A. Crespo, Yunrong Li, Javier Ruiz–Castillo

The second author's name was spelled incorrectly in the author byline.

The correct name is: Yunrong Li.

